# The impact of traumatic childhood experiences on interoception: disregarding one’s own body

**DOI:** 10.1186/s40479-023-00212-5

**Published:** 2023-02-15

**Authors:** Marius Schmitz, Sarah N. Back, Katja I. Seitz, Nele K. Harbrecht, Lena Streckert, André Schulz, Sabine C. Herpertz, Katja Bertsch

**Affiliations:** 1 Department of General Psychiatry, Center for Psychosocial Medicine, University of Heidelberg, Heidelberg, Germany; 2 Department of Psychology, Ludwig-Maximilians-University Munich, Munich, Germany; 3 Institute of Psychology, University of Heidelberg, Heidelberg, Germany; 4 Department of Behavioural and Cognitive Sciences, Clinical Psychophysiology Laboratory, University of Luxembourg, Esch-Sur-Alzette, Luxembourg

**Keywords:** Dissociation, Stress, Trauma, Emotion dysregulation, Beliefs, Interoception

## Abstract

**Background:**

Deficient interoception, the processing and perception of internal bodily signals, has been discussed as a mechanism underlying various mental disorders. First results indicate a mediating role of interoception in the interplay of traumatic childhood experiences and adult mental disorders. Traumatic childhood experiences may hinder the adequate processing, integration, and trust in bodily signals that are important in order to understand and regulate own needs and emotions, thereby increasing the vulnerability for mental disorders. However, an overarching study investigating alterations in different interoceptive measures and trauma-related disorders as well as their mediating role between early trauma and emotion dysregulation is still missing.

**Methods:**

One hundred thirty-six individuals with varying levels of traumatic childhood experiences who either had a current diagnosis of major depression, posttraumatic stress disorder, or somatic symptom disorder, or no mental disorder, took part in a multidimensional assessment of interoceptive processes, including interoceptive accuracy, sensibility, and awareness. Kruskal–Wallis tests were used to compare groups regarding interoceptive processes and associations with traumatic childhood experiences and emotion dysregulation were analyzed with Spearman correlations. Furthermore, mediation analyses were computed to examine and compare interoceptive processes as potential mediators between traumatic childhood experiences and emotion dysregulation.

**Results:**

Only body dissociation, a measure for interoceptive sensibility, was significantly reduced in individuals with a current mental disorder. Body dissociation was also the only interoceptive measure significantly associated with traumatic childhood experiences and emotion dysregulation and the only significant mediator in the relationship between traumatic childhood experiences and emotion dysregulation across groups.

**Conclusion:**

Results suggest body dissociation, but not other interoceptive measures, as an important feature linking traumatic childhood experiences to current emotion dysregulation, an important transdiagnostic feature. As body dissociation refers to a habitual non-attendance or disregard of interoceptive signals, integrative therapeutic interventions could help affected individuals to overcome difficulties in emotion perception and regulation.

**Trial registration:**

The general study design was preregistered; see the German Clinical Trials Register (DRKS-ID: DRKS00015182). This study’s analysis plan was not preregistered.

**Supplementary Information:**

The online version contains supplementary material available at 10.1186/s40479-023-00212-5.

## Background

Traumatic childhood experiences (TCEs) are an important singular risk factor for mental disorders later in life [1–3]. TCEs include a variety of adverse experiences in childhood, including emotional or physical neglect as well as emotional, physical, and sexual abuse [4]. Despite tremendous progress, the mechanisms and pathways by which TCEs lead to later psychopatholgy are still not fully clear [5–8]. One possible mediator for the observed link between TCEs and psychopathology might be alterations in interoception, i.e. the processing and perception of signals from inside the body [9].

Ranging from afferent signal transmission and its cortical representation to conscious perception of own bodily signals [10, 11], interoceptive processes constitute a multifaceted system. Three important facets include (1) conscious interoceptive processes, which can be operationalised by the correspondence between perceived and actual body signal, e.g., assessed using heartbeat perception tasks [12] and are referred to as interoceptive accuracy, (2) self-reported evaluation of one’s own interoceptive abilities which is known as interoceptive sensibility, and (3) meta-cognitive interoceptive awareness, i.e., the convergence between interoceptive accuracy and sensibility [13].

It has been assumed that interoceptive processes play a major role for mental health [9]. Moreover, it has recently been suggested that TCEs, besides a biological vulnerability, might lead to impaired interoception and thereby to reduced emotional awareness and heightened emotion dysregulation, such as in borderline personality disorder (BPD) [14]. The importance of interoception for emotion perception and regulation has been stated since early emotion theories [15–20]. Interoception includes both the ability and willingness to attend to own inner processes, feelings, and needs. Based on the active inference framework [21–24], interoceptive deficits have been proposed to occur when afferent interoceptive evidence is no longer integrated in inner models of bodily states. The discrepancy between predicted bodily state and interoceptive evidence results in the emergence of error signals. In the case of adaptive functioning, such error signals are resolved by updating inner models and used to informing actions to (re-)maintain homeostasis [25]. However, inadequate adjustment can lead to a persistent presence of error signals. This may either be due to (1) the afferent signalling itself, such as weak or unprecise interoceptive signals, (2) overly strong inner models, or (3) context rigidity, i.e., the same inner model is maintained even when the context changes. Since interoception is important for homeostasis [26, 27] and emotion regulation [28, 29], with emotion regulation representing strategies to (re-)maintain emotional homeostasis and body-related symptoms, deficient interoception might represent a possible mediating pathway between TCEs and emotion dysregulation.

Indeed, there is first evidence for this assumption. First, alterations in interoceptive processes have been reported in trauma-related disorders, which also show deficits in emotion regulation. Such disorders encompass, but are not limited to, posttraumatic stress disorder (PTSD), BPD, major depression (MD), and somatic symptom disorder (SSD), all of which show high prevalence of TCEs [30–36] and exhibit emotion regulation deficits [37–41]. Interoceptive impairments which have been investigated so far yielded inconsistent results of reduced to normal interoceptive signal processing [42–44], as well as reduced to normal interoceptive accuracy [45–48] and reduced interoceptive sensibility [49, 50]. However, it must be noted that, up to now, not all interoceptive dimensions have been studied in all presented disorders. Second, first studies suggest an association between TCEs and interoceptive processes. Interoceptive accuracy and TCEs were negatively associated after an acute stressor in healthy individuals [51] and sympatho-adreno-medullary (SAM) axis activation led to decreased interoceptive accuracy in healthy individuals with TCEs [52]. Third, a first mediation model in women with BPD showed that body dissociation, a measure for interoceptive sensibility, mediated the link between TCEs and emotion dysregulation in patients with BPD [50]. However, it remains unclear whether altered interoception is limited to trauma-related mental disorders or represents a transdiagnostic feature related to TCEs [29]. This differentiation is crucial to develop new diagnostic and treatment approaches targeting interoceptive dysfunctions.

The current study sought to close this gap. Following a multidimensional conceptualization of interoception [10, 13], we assessed interoceptive accuracy, sensibility, and awareness in a relatively large well-characterized sample of patients with MD, PTSD, or SSD as well as healthy controls with varying levels of TCEs. Additionally, we measured heart rate variability (HRV), i.e., the beat-to-beat variability of heart rate, which represents an index of cardiac control through the autonomic nervous system (ANS) [53] and actual sympathetic and parasympathetic output [54], which has been shown to be reduced in trauma-related disorders [55] and associated with interoceptive processing [56–59].

The aims of the study were threefold. First, we investigated alterations in interoceptive processes among three different mental disorders and healthy individuals. Second, we investigated associations between TCEs, interoceptive processes, and emotion dysregulation across diagnostic categories. Finally, we studied the proposed mediating role of interoceptive processes between TCEs and emotion dysregulation as an important transdiagnostic feature.

Based on previous studies, we expected to replicate interoceptive impairments in trauma-related disorders and explored the specificity of previous findings compared to clinical controls. Moreover, since previous studies included heterogenous samples of patients with and without TCEs, we were able to further inspect the impact of TCEs on interoceptive processes. We expected negative associations between interoceptive processes and both TCEs as well as emotion dysregulation, and that interoceptive processes significantly mediate the relationship between TCEs and emotion dysregulation across groups.

## Methods

### Design

This research was part of a larger study on the shared effects of TCEs on social information processing across different mental disorders with high prevalence of TCEs, consisting of patients with MD, PTSD, and SSD, as well as HC (German Clinical Trials Register: DRKS00015182). Therefore, a multiple-group cross-sectional design was employed, including participants with varying levels of TCEs following a further dimensional conceptualization. The original study is part of the German Research Foundation’s Research Training Group 2350, dedicated to investigating the impact of adverse childhood experiences on psychosocial and somatic conditions across the life span [60]. All participants gave written informed consent before their participation and were reimbursed for their participation. The study was approved by the ethics review board of the Medical Faculty Mannheim, Heidelberg University.

### Recruitment and enrollment

Participants were recruited from online announcements, flyers, and through a clinical referral from inpatient and outpatient departments. All participants were fluent in the German language.

#### Inclusion and exclusion criteria

General exclusion criteria for all participants were (a) age under 18 years or over 60 years; (b) neurological disorders; (c) current substance abuse, assessed via urine toxicology screening and clinical interview; (d) severe medical illness; (e) pregnancy, and (f) left-handedness due to fMRI measurements (not reported here). Additional general exclusion criteria for participants with mental disorders were lifetime diagnoses of schizophrenia, schizoaffective, or bipolar disorder and severe substance use disorder in the last two years. Inclusion of psychotropic medications for participants with mental disorders were limited to regularly prescribed antidepressants, antipsychotics (sleep-inducing effect only), and/or anticonvulsants (i.e., pregabalin, pain-relieving effect only) (see Table S1 in the Supplement).

*Participants with mental disorders* had to fulfill diagnostic criteria for a diagnosis of PTSD, MD, or SSD, with diagnostic group allocation based on the current diagnosis that had been made first during the participant’s lifetime. Therefore, participants could be diagnosed with up to three of these disorders of interest (i.e., MD, PTSD, SSD), but were excluded if the current diagnosis was not determined as first lifetime disorder. Mental disorders were assessed with the Structured Clinical Interview for DSM-5 (SCID-5) [61].

The inclusion criterion for *healthy controls (HC)* was the absence of any mental disorder, either current or lifetime, as assessed using the SCID-5.

Out of 140 adult participants, four individuals were excluded from the current analysis due to cardiac arrhythmia (*n* = 1) or missing of both behavioral and self-reported data on interoception (technical problems and non collecting self-reports, *n* = 2; dropout due to aberrant neurological finding, *n* = 1), resulting in a final sample of 136 participants (see Table 1 and Table S1 in the Supplement for details). All participants experienced at least one TCE and were classified into the four diagnostic groups MD (*N* = 35 [24 female], *M*_age_ = 31.74, *SD* = 12.09 years), SSD (*N* = 34 [26 female], M_age_ = 30.09, 11.59 years), PTSD (*N* = 33 [28 female], M_age_ = 34.33, *SD* = 12.48 years), and HC (*N* = 34 [27 female], M_age_ = 29.56, *SD* = 9.64 years). The groups did not differ in age (*F*_3,132_ = 1.17, *p* = 0.326), body-mass-index (*F*_3,132_ = 1.23, *p* = 0.303), highest school degree (Kruskal–Wallis test: *H*[3] = 5.41, *p* = 0.144), or sex distribution (*χ*^2^_df=3_ = 2.68, *p* = 0.447).

**Table 1:** Sample Characteristics

**Data**	**Mean ± SD** **(Mdn; IQR)**				***H* Value**	***p* Value**^***a***^	***p* Value**^***b***^					
	**MD** **(*n* = 35)** **(24 women)**	**SSD** **(*n* = 34)** **(26 female)**	**PTSD** **(*n* = 33)** **(28 female)**	**HC** **(*n* = 34)** **(27 female)**			***MD*** ***vs*** ***SSD***	***MD*** ***vs*** ***PTSD***	***MD*** ***vs*** ***HC***	***SSD*** ***vs*** ***PTSD***	***SSD*** ***vs*** ***HC***	***PTSD*** ***vs*** ***HC***
**Depression severity** **(BDI-II)**^**c**^	32.74 ± 9.65 (31.00; 18.00)	16.26 ± 9.49 (17.50; 17.75)	25.33 ± 11.57 (25.00; 19.00)	5.12 ± 4.52 (4.00; 8.25)	78.96	< .001*	< .001*	.283	< .001*	.039*	.002*	< .001*
**SSD-12**^**c**^	18.46 ± 10.91 (18.00; 16.00)	30.56 ± 6.58 (31.00; 8.25)	19.52 ± 9.81 (20.00; 13.00)	7.91 ± 8.07 (5.00; 11.00)	60.90	< .001*	< .001*	1.00	.001*	.001*	< .001*	< .001*
**PHQ-15**^**c**^	11.43 ± 5.00 (11.00; 6.00)	12.62 ± 4.79 (13.00;6.00)	12.45 ± 5.53 (13.00; 7.00)	6.65 ± 5.07 (6.00; 7.50)	25.10	< .001*	1.00	1.00	.007*	1.00	< .001*	< .001*
**PCL-5**	27.46 ± 19.13 (29.00; 32.00)	17.03 ± 15.29 (12.50; 17.25)	41.36 ± 13.16 (41.00; 22.00)	8.56 ± 11.01 (3.50; 12.00)	55.68	< .001*	.160	.027*	< .001*	< .001*	.200	< .001*
**Early Traumatization** **(CTQ)**	47.34 ± 13.46 (45.00; 18.00)	40.79 ± 12.37 (37.50; 17.25)	69.82 ± 22.84 (71.00; 33.00)	47.65 ± 17.95 (44.00; 28.50)	33.69	< .001*	.401	.001*	1.00	< .001*	.893	< .001*
Emotional Abuse	12.91 ± 5.46 (13.00; 9.00)	10.29 ± 4.71 (9.00; 5.25)	17.97 ± 6.20 (20.00; 11.00)	12.18 ± 5.71 (11.00; 10.50)	25.57	< .001*	.340	.015*	1.00	< .001*	1.00	.002*
Physical Abuse	7.20 ± 2.92 (6.00; 3.00)	6.56 ± 2.96 (5.00; 2.25)	11.94 ± 6.10 (10.00; 9.50)	9.09 ± 5.21 (7.50; 6.00)	26.22	< .001*	1.00	< .001*	1.00	< .001*	.062	.115
Sexual Abuse	6.17 ± 2.92 (5.00; 1.00)	5.59 ± 2.06 (5.00; 0.00)	10.85 ± 6.50 (9.00; 8.00)	6.18 ± 2.90 (5.00; 0.00)	35.65	< .001*	1.00	< .001*	1.00	< .001*	1.00	< .001*
Emotional Neglect	13.74 ± 5.34 (13.00; 6.00)	11.44 ± 5.05 (11.00; 8.00)	18.06 ± 5.53 (20.00; 9.00)	12.38 ± 4.93 (12.00; 9.00)	23.96	< .001*	.545	.020*	1.00	< .001*	1.00	.001*
Physical Neglect	7.31 ± 2.61 (7.00; 3.00)	6.91 ± 2.37 (6.00; 4.00)	11.00 ± 4.58 (11.00; 8.00)	7.82 ± 3.13 (7.00; 4.25)	20.47	< .001*	1.00	.003*	1.00	< .001*	1.00	.020*
**DERS**^**c**^	116.60 ± 22.68 (117.00; 32.00)	99.09 ± 20.82 (97.50.00; 29.00)	116.39 ± 22.04 (119.00; 28.50)	78.79 ± 19.66 (80.00; 25.00)	47.76	< .001*	.025*	1.00	< .001*	.024*	.014*	< .001*
**FDS**^**d**^	13.95 ± 9.91 (12.50; 12.05)	10.81 ± 8.22 (7.73; 10.51)	16.73 ± 12.22 (12.27; 10.68)	6.39 ± 6.28 (4.55; 6.19)	24.62	< .001*	1.00	1.00	.001*	.187	.074	< .001*
**BSI**	1.41 ± 0.53 (1.36; 0.51)	0.80 ± 0.42 (0.75; 0.78)	1.26 ± 0.67 (1.17; 1.19)	0.31 ± 0.35 (0.22; 0.15)	66.87	< .001*	.002*	1.00	< .001*	.059	.001*	< .001*

Significant values are highlighted via asterisk

*Abbreviations: BDI-II* Beck Depression Inventory revised, *BS*I Brief Symptom Inventory, *CTQ* Childhood Trauma Questionniare, *DERS* Difficulties in Emotion Regulation Scale, *FDS* German adaptation of the Dissociative Experience Scale (DES), *H* test statistic of the Kruskal–Wallis test, *HC* Healthy controls, *IQR* Interquartil range, *MD* Major depressive disorder, *Mdn* Median, *p Value* Probability value, *PCL-5* PTSD Checklist for DSM-5, *PTSD* Posttraumatic stress disorder, *PHQ-15* Patient Health-Questionnaire-15, *SD* standard deviation, *SSD* somatic symptom disorder, *SSD-12* Somatic Symptom Disorder B-Criteria Scale

^*a*^Uncorrected for multiple testing

^*b*^Corrected for multiple testing via Bonferonni

^c^*n* = 2 missings replaced by group mean values

^d^*n* = 3 missings replaced by group mean values

## Materials and Methods

In this section, measures of all relevant constructs are presented. For details, please refer to the Supplement.

### Traumatic childhood experiences

*TCEs* were assessed with the *Childhood Trauma Questionnaire* (CTQ) [62]. The CTQ measures physical, sexual, and emotional abuse as well as physical and emotional neglect. A total sum score was calculated, ranging from 25 to 125, with higher values indicating a higher frequency of traumatic experiences.

### Psychopathology

Mental health disorders were assessed using the *Structured Clinical Interview for DSM-5* (SCID-5) [61] (Interrater reliability: κ = 1.00). The severity of common somatic symptoms was assessed using the *Patient Health-Questionnaire-15* (PHQ-15) [63] and the *Somatic Symptom Disorder—B Criteria Scale* (SSD-12) [64] was used to assess SSD symptomatology. PTSD symptom severity was assessed using the *Posttraumatic Stress Disorder Checklist for DSM-5* (PCL-5) [65]. Severity of depressive symptoms was assessed with the *Beck-Depression-Inventory II* (BDI-II) [66]. General symptom severity was assessed with the *Brief Symptom Inventory* (BSI) [67]. Due to its overlap with body dissociation, a measure of trait dissociation was administered in order to investigate the specifity of body dissociation in the current study [50]. Thus, the German adaptation of the *Dissociative Experience Scale*, the *Fragebogen zur Erfassung Dissoziativer Symptome* (FDS) [68, 69] was used.

### Emotion dysregulation

Emotion regulation deficits were assessed with the *Difficulties in Emotion Regulation Scale* (DERS) [70]. The DERS comprises six subscales: *nonacceptance of negative emotions*, *difficulties engaging in goal-directed behaviors when distressed*, *difficulties controlling impulsive behaviors when distressed*, *limited access to effective emotion regulation strategies*, *lack of emotional awareness*, and *lack of emotional clarity*. A total sum score was calculated, ranging from 36 to 180, with higher values indicating more severe deficits in emotion regulation.

### Interoceptive processes

*Interoceptive sensibility* was measured both via self-reported interoceptive task-confidence and self-report questionnaire. The mean score of the confidence ratings across heartbeat counting trials was calculated as a global measure of interoceptive sensibility pertaining to self-reported heartbeat perception [13]. The *Scale of Body Connection* (SBC) [71] was used to assess self-reported *body awareness* and *body dissociation* during the last two months. *Body awareness* measures attention to bodily signals in everyday situations and the perception of bodily responses to emotions. *Body dissociation* refers to the avoidance or disregard of internal bodily experiences and the feeling of seperatedness from one’s own body. Mean scores, ranging from 0 to 4, were calculated for each scale, with higher values indicating higher body awareness and body dissociation, respectively.

*Interoceptive accuracy* was assessed by means of the heartbeat counting task [72]. A heartbeat perception score was calculated, across seven consecutive time intervals of varying length unknown to the participants (20, 25, 35, 45, 55, 65, 75 s), by comparing the perceived number of heartbeats (HB) and the actual number of heartbeats, with higher values (maximum of 1) indicating higher interoceptive accuracy (overall internal consistency α = 0.96) [73].

$$\mathrm{IAc}=\frac{1}{7}\sum_{k=1}^{7}\begin{array}{c}1- \frac{\left|\sum {\mathrm{HB actual}}_{k}- \sum {\mathrm{HB perceived}}_{k}\right|}{\sum {\mathrm{HB actual}}_{k}}\\ \end{array}$$

*Interoceptive awareness* was calculated as the within-participant Pearson correlation *r* [13], between interoceptive accuracy and confidence averaged across trials, resulting in an interoceptive awareness score ranging from -1 to + 1. Negative values indicate a discrepancy between confidence and objective interoceptive accuracy, while positive values indicate an accordance and values near zero indicate low interoceptive awareness:

$$\frac{\frac{{\sum }_{i=participant}^{\mathrm{N}=7}({x}_{in=trial}- {\overline{x} }_{i})({y}_{in=rating}- {\overline{y} }_{i})}{N-1}}{s\left({x}_{i}\right)*s({y}_{i})}$$

HRV was operationalised using the root-mean-square of successive R–R-interval differences (RMSSD). RMSSD was chosen as it is claimed to be a comparably robust and statistically reliable indicator of vagally-mediated short-term HRV [74], which is mostly unaffected by breathing artefacts [75].

### Procedure

Participants completed a 5-min resting-state electrocardiogram (ECG) measurement before performing the heartbeat counting task. The ECG was recorded using Einthoven II electrode placement. HRV-Analysis was based on resting-state RMSSD values as HRV index. For details, see the Supplement.

### Statistical analysis

Analyses were performed using IBM SPSS v26.0 (descriptives and correlation analyses) and R v3.5.0 via R plug-in for SPSS (mediation analysis). To account for deviations from normality, non-parametric analyses were performed. Two-tailed *p* < 0.05 was employed for all analyses.

#### Aim 1: Group comparisons for interoceptive processes

Kruskal–Wallis tests were used to compare groups regarding interoceptive measures. Dunn-Bonferroni-tests were conducted as post-hoc tests following significant effects (r as effect size) [76].

#### Aim 2: Correlation analysis between TCEs, interoceptive dimensions and emotion dysregulation

Spearman correlations were used to investigate the relationship between interoceptive dimensions and both TCEs and emotion dysregulation for the whole sample.

#### Aim 3: Analysis of the mediating role of interoceptive processes between TCEs and emotion dysregulation

Mediation analysis was performed using the ROBMED macro with robust bootstrap for SPSS (v0.6.0; bootstrapping procedure: 10,000 samples, confidence intervals: 95%, unstandardized coefficients, adjusted robust R^2^ as effect size) (Alfons et al.: A Robust Bootstrap Test for Mediation Analysis (August 3, 2018), forthcoming). The mediation model included interoceptive accuracy (heartbeat counting task), interoceptive sensibility (mean confidence, body awareness, body dissociation), interoceptive awareness, and RMSSD as HRV index. Only complete cases (*N* = 99) were included (missings: *n* = 26 technical recording issues, *n* = 9 missing self-report, *n* = 2 physiological aberrant finding, *n* = 1 task difficulties).

## Results

### Group comparisons for interoceptive processes

There was a significant group difference in all three measures of interoceptive sensibility (see Table 2): Patients with MD had significantly lower levels of mean confidence than HC (effect size *r* = -0.37), patients with SSD had significantly higher levels of body awareness than patients with MD and patients with PTSD (MD: *r* = 0.33, PTSD: *r* = 0.46), and all three patient groups reported significantly higher body dissociation than HC (MD: *r* = 0.44, SSD: *r* = 0.37, PTSD: *r* = 0.68). However, groups did not differ significantly in interoceptive accuracy or interoceptive awareness. With regard to HRV, patients with MD showed lower RMSSD as HRV index, compared to patients with SSD (*r* = -0.40) and HC (*r* = -0.45). The groups did not differ in heart rate (*H*[3] = 0.58, *p* = 0.901).

**Table 2:** Sample Characteristics for Interoceptive Dimensions

**Interoceptive dimensions**	**Mean ± SD** **(Mdn; IQR)**				***H* Value**	***p* Value**^***a***^	***p* Value**^***b***^					
	**MD**	**SSD**	**PTSD**	**HC**			***MD*** ***vs*** ***SSD***	***MD*** ***vs*** ***PTSD***	***MD*** ***vs*** ***HC***	***SSD*** ***vs*** ***PTSD***	***SSD*** ***vs*** ***HC***	***PTSD*** ***vs*** ***HC***
**Interoceptive accuracy**	0.65 ± 0.19 (0.70; 0.33)	0.60 ± 0.18 (0.60; 0.30)	0.62 ± 0.16 (0.61; 0.23)	0.62 ± 0.26 (0.66; 0.26)	1.39	.708						
**Confidence**	3.64 ± 1.87 (2.86; 1.93)	4.35 ± 1.54 (4.00; 2.89)	4.02 ± 2.08 (3.21; 3.18)	4.94 ± 1.75 (5.14; 3.00)	9.57	.023*	.446	1.00	.018*	1.00	1.00	.308
**Interoceptive awareness**	0.17 ± 0.39 (0.19; 0.52)	0.26 ± 0.44 (0.40; 0.68)	0.21 ± 0.42 (0.18; 0.65)	0.41 ± 0.43 (0.51; 0.89)	6.80	.079						
**Body Awareness** **(SBC)**	2.40 ± 0.53 (2.38; 0.56)	2.75 ± 0.52 (2.83; 0.65)	2.15 ± 0.68 (2.33; 0.83)	2.46 ± 0.57 (2.50; 0.92)	14.87	.002*	.046*	1.00	1.00	.001*	.275	.706
**Body Dissociation (SBC)**	1.45 ± 0.65 (1.25; 0.75)	1.33 ± 0.59 (1.38; 0.88)	1.82 ± 0.69 (1.87; 1.09)	0.82 ± 0.63 (0.63; 1.00)	27.53	< .001*	1.00	.379	.004*	.089	.022*	. < 001*
**HRV (RMSSD)**	27.77 ± 19.22 (20.70; 13.69)	40.73 ± 20.33 (33.57; 25.67)	32.54 ± 15.43 (28.17; 20.13)	49.21 ± 30.92 (44.48; 53.25)	16.22	.001*	.010*	.667	.002*	.730	1.00	.269

Significant values are highlighted via asterisk

*Abbreviations: H* test statistic of the Kruskal–Wallis test, *HC* Healthy controls, *HRV* Heart rate variability, *IQR* Interquartil range, MD major depressive disorder, *Mdn* Median, *p Value* Probability value, *PTSD* Posttraumatic stress disorder, *RMSSD* Root Mean Square of Successive Differences, *SBC* Scale of Body Connection, *SD* Standard deviation, *SSD* Somatic symptom disorder

^*a*^Uncorrected for multiple testing

^*b*^Corrected for multiple testing via Bonferonni

### Correlation analysis between TCEs, interoceptive dimensions and emotion dysregulation

Both TCEs (CTQ score) and emotion dysregulation (DERS score) were positively correlated with self-reported body dissociation (see Table 3). No further significant correlations were obtained for the remaining interoceptive measures after controlling for multiple testing.

**Table 3:** Associations between traumatic childhood experiences, emotion dysregulation, and interoceptive dimensions

	**Traumatic childhood experiences**							
Traumatic childhood experiences (CTQ)	1	**Emotion Dysregulation**						
Emotion Dysregulation (DERS)	.333* < .001 *N* = 136	1	**Interoceptive accuracy**					
Interoceptive accuracy	.073 .437 *N* = 116	.023 .805 *N* = 116	1	**Confidence**				
Confidence	-.196 .035 *N* = 116	-.170 .068 *N* = 116	-.005 .956 *N* = 116	1	**Awareness**			
Awareness	-.098 .298 *N* = 116	-.198 .033 *N* = 116	-.211 .023 *N* = 116	.069 .463 *N* = 116	1	**Body Awareness (SBC)**		
Body Awareness (SBC)	-.203 .024 *N* = 124	-.209 .020 *N* = 124	-.281* .004 *N* = 105	.261 .007 *N* = 105	.042 .674 *N* = 105	1	**Body Dissociation (SBC)**	
Body Dissociation (SBC)	.372* < .001 *N* = 124	.555* < .001 *N* = 124	.007 .940 *N* = 105	-.216 .027 *N* = 105	-.194 .048 *N* = 105	-.199 .027 *N* = 124	1	**HRV (RMSSD)**
HRV (RMSSD)	-.077 .392 *N* = 126	**-**.211 .018 *N* = 126	.170 .076 *N* = 110	-.094 .330 *N* = 110	.159 .097 *N* = 110	-.024 .803 *N* = 114	-.310* .001 *N* = 114	1

Spearman correlations are presented in the first row of each cell, followed by the probability value and sample size. Critical alpha values are set to (0.05/8 = .006) with significant values highlighted via asterisk

*Abbreviations: CTQ* Childhood Trauma Questionniare, *DERS* Difficulties in Emotion Regulation Scale, *HRV* Heart rate variability, R*MSSD* Root Mean Square of Successive Differences, *SBC* Scale of Body Connection

### Analysis of the mediating role of interoceptive processes between TCEs and emotion dysregulation

**Fig. 1:**
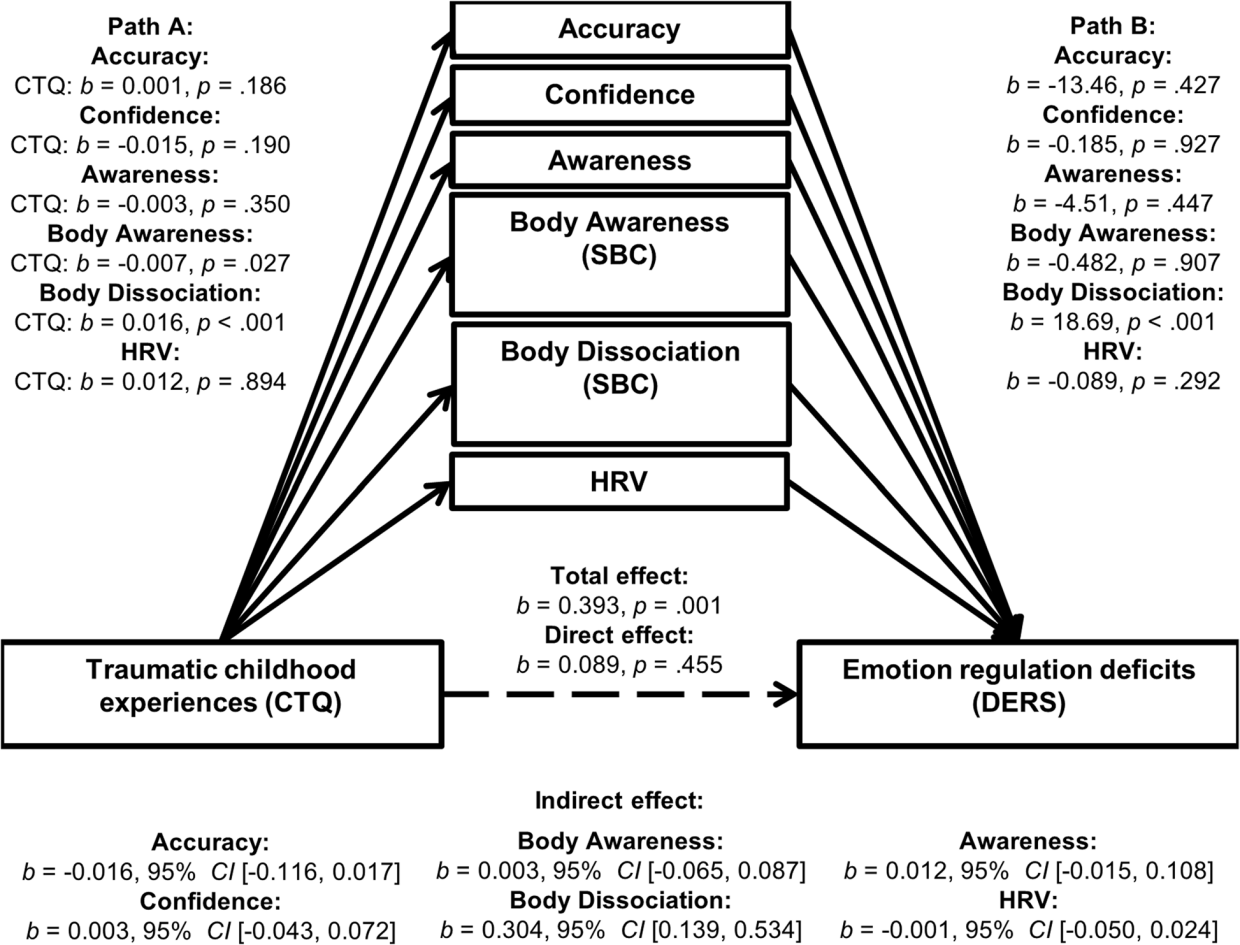
Parallel Mediation Model in a Sample of Patients with Major Depression, Somatic Symptom Disorder, Posttraumatic Stress Disorder, and Healthy Controls (*N* = 99). Path A represents the effect of the predictor on each mediator, path B represents the combined relationship of each mediator with the outcome, with the direct effect representing the effect of the predictor on the outcome after inclusion of all mediators and the total effect representing the basic relationship between the predictor and the outcome. The indirect effect represents the combined effect of path A and path B and therefore the mediation. Significance inferences at the 0.05 α level are based upon the notion whether confidence intervals include zero. *Abbreviations.* CTQ, Childhood Trauma Questionnaire; DERS, Difficulties in Emotion Regulation Scale; HRV, heart rate variability; SBC; Scale of Body Connection

The mediation analysis revealed a significant indirect effect of TCEs (CTQ total score) on emotion dysregulation (DERS total score) through body dissociation (*b* = 0.304, 95% CI [0.139, 0.534]), including interoceptive accuracy, confidence ratings, interoceptive awareness, body awareness, and HRV (RMSSD) as parallel mediators (see Fig. 1). While the total effect of TCEs on emotion dysregulation was significant (*b* = 0.393 *p* = 0.001), the direct effect was not after including the mediators (*b* = 0.089, *p* = 0.455, adjusted robust *R*^2^ = 0.325). In the current mediation model, body dissociation, but not the other interoceptive measures, mediated the association between TCEs and emotion dysregulation.

The observed pattern remained even when trait dissociation was entered as a parallel mediator as can be seen in the Supplement (see Fig. S1).

## Discussion

In the current study, we investigated whether interoceptive processes are altered in disorders related to TCEs, are associated with TCEs and emotion dysregulation, and do mediate the relationship between TCEs and emotion dysregulation, an important transdiagnostic feature. Interestingly, in partial support of aim 1, we found consistent alterations in only one measure of interoceptive sensibility, namely body dissociation with higher scores in patients with PTSD, SSD, and MD compared to healthy controls. Furthermore, with regard to aims 2 and 3, body dissociation also appeared to be the only measure significantly associated with TCEs as well as significant mediator of the association between TCEs and emotion dysregulation in this large and well-characterized sample of individuals with varying TCEs.

Our findings of higher body dissociation in three different groups of patients with trauma-related disorders as well as positive associations to TCEs and emotion dysregulation are in line with earlier studies showing higher body dissociation in individuals with a history of TCEs [71, 77]. Likewise, the observed associations correspond to similar findings of heightened dissociation [78–80], a factor that we controlled for in our analysis. Importantly, body dissociation, a measure of reduced or impaired interoceptive sensibility in terms of habitual disregard or non-attendance of interoceptive signals [81, 82], was found to mediate the association between TCEs and emotion dysregulation amongst all measured interoceptive processes. The current finding replicates and expands an earlier mediation analysis in women with BPD [50].

An explanation for its putative clinical importance in individuals with TCEs and mediating role might be that body dissociation represents an inner attitude toward one`s own body and a non-adaptive emotion regulation attempt due to TCEs. Whereas interoceptive signal transmission seems to be sufficient during an attention state, such as a heartbeat counting task, body dissociation might indicate a coping style. A habitual avoidance or disregard of internal bodily experiences and the feeling of seperatedness from one’s own body might reflect a focus on external stimuli as a protective strategy due to a history of TCEs. This kind of strategy may have important implications. First, individuals with TCEs and higher body dissociation might not be able to accurately detect and monitor bodily signals without a conscious state of attention in everyday life. Second, individuals with TCEs might have learned to mistrust their own bodily signals, either because they regard them as dangerous or simply as unhelpful for determining one’s own emotional state, and therefore choose to disregard them. Third, individuals with TCEs might have difficulties integrating and using bodily signals as internal cues for their own emotions and needs. Given the importance of bodily signals for homeostasis [26, 27] and emotion regulation [28, 29], body dissociation might represent a clinical variable of interest for psychological interventions. Furthermore, it highlights the need to assess interoceptive processes more closely in everyday life in order to be able to determine and disentangle the causes and mechanisms underlying heightened body dissociation in individuals with TCEs.

One such mechanism might be stress. Stress responses form a complex neuro-behavioral cascade, which includes physiological changes and corresponding physical symptoms [83]. While acute stress reflects a response to a potentially harmful stimulus of limited duration, chronic stress can be elicited either by prolonged exposure or perpetuated in the aftermath of severe stressors. Both acute and chronic stress have been shown to impact interoceptive processes [83]. Of note, interoceptive accuracy and TCEs have been shown to be only associated during states of acute stress [51, 52]. Chronic stress as experienced through TCEs might induce malfunctions in the body-brain communication which become prominent in states of acute stress, wherein deficient processing of physical symptoms might hinder regulative processes [83, 84]. One might speculate that the recurrence of such experiences and failed adaptive regulations leads to persistent internal error signals which in turn may lead to heightened body dissociation. Whether body dissociation alters the perception of interoceptive signals during acute stress needs to be investigated in further studies. Error signals should become prominent during acute stress, in an attempt to restore homeostasis instead of a general background noise of interoceptive dysfunction. Such error signals might even replace habitual disregard of bodily symptoms by perceptions of physical symptoms in a positive feedback loop [85], thereby representing interoceptive regulation attempts [84]. Therefore, alterations in some interoceptive processes might only to be expected during homeostatic perturbations [86] such as acute stress [51, 52, 87]. Whether further interoceptive processes might emerge as mediators during active emotion regulation demands in individuals with TCEs needs to be investigated in future studies.

Although interoceptive accuracy has been studied intensively, we did not find neither a mediation effect nor significant group differences at rest in the current sample. Of note, potential methodological shortcomings of the heartbeat counting task have been debated in the literature, some of which may compromise its validity. One account is that the original task is contaminated by non-interoceptive processes, such as estimating one’s own heartbeats and under-reporting [88]. However, in order to be able to compare current findings with previous studies, the original setup of the heartbeat counting task was administered, resulting in a ratio of 13.79% of of ‘good’ heartbeat perceivers (based on a score greater than 0.85) [89], a finding which has has been reported in previous studies [57, 90]. Although, average interoceptive confidence and interoceptive accuracy were not significantly associated, all groups showed on average positive values of interoceptive awareness, suggesting that most participants were able to judge their actual accuracy in the heartbeat counting task. Therefore, we conclude that the participants were able to form metacognitive beliefs, which corresponded on average to the achieved task performance. However, the current mediation analysis and group comparisons suggest that the heartbeat counting task and measures building upon it (i.e., confidence ratings and interoceptive awareness), did not contribute to reveal interoceptive deficits and mediators in the current sample. Similarly, performance on the heartbeat counting task did not show associations to mental health outcomes in a recent meta-analysis [91], further suggesting that other operationalisations of interoceptive accuracy might be more suitable to reveal interoceptive approaches to treatment [92].

Likewise, RMSSD as HRV index did not emerge as a significant mediator in our current mediation analysis. The lacking finding of a direct link between TCEs and HRV is in line with a recent meta-analysis [93]. HRV, as indexed by RMSSD, represents both parasympathetic tone [94], which can serve as an indicator of cardiac activation and afferent bodily signal strength [95], as well as cardiac adaptability and control. Lower HRV has been shown to be related to adverse physical health outcomes [54] and found to be typically reduced in trauma-related disorders [55]. The current findings suggest that parasympathetic regulation does not necessarily play a major role for the link between TCEs and emotion dysregulation. This was unexpected, since dysfunctions of the ANS, as reflected by altered HRV, have been associated with stress [96]. In contrast, significantly lower RMSSD was found only in the MD group. Although not statistically significant, the observed pattern of lower HRV in the three patient groups are in line with previous research [55] indicating, on average, a tendency of autonomic dysregulation in the trauma-related patient groups. Of note, the HC group showed a relatively high standard deviation for the RMSSD which might have masked further group differences. Although RMSSD as HRV index was not correlated with TCEs, the finding of a high variance in the HC group [97] characterized by TCEs needs future investigation. Interestingly, RMSSD as HRV index in the current study was negatively correlated with body dissociation but uncorrelated with interoceptive accuracy. The latter finding is inconsistent with a previous study [57], wherein a positive relationship was found. However, further studies with higher sample sizes are needed in order to disentangle symptom severity, psychotropic medication load, and HRV, and to control for possible confounding variables [98] before strong conclusions can be drawn. In addition, as HRV has been linked to dissociative experiences [99, 100], associations between HRV and body dissociation need to be further examined in the future.

Of importance, the current study revealed interoceptive deficits in interoceptive sensibility within the sample of trauma-related disorders. Whereas patients with SSD tended to exhibit higher body awareness, which might be indicative of an habitual attention tendency as reflected in the SBC [82] and could interact with bodily distress [101], patients with MD reported lower levels of mean confidence. Although this finding needs further replication especially in moderately depressed patients [47, 102], patients with MD might show a general tendency of lower task performance confidence but are able to adequatly judge their performance in a trial-by-trial evaluation. Patients with PTSD showed higher body dissociation alongside the other two clinical groups. As body awareness was not significantly altered in patients with PTSD, it needs to be further examined whether heightened body dissociation might be interpreted as a form of experiental avoidance in PTSD.

In summary, the findings of the current study underline the importance of interoceptive sensibility and metacognitive beliefs such as the disregard of one’s bodily signals due to body dissociation. The results are in line with the notion that physiological interoceptive states and interoceptive accuracy, as mostly measured in interoceptive studies, might not sufficiently capture relevant (higher-order) interoceptive processes [10, 13]. As outlined by [103] in their 2 × 2 factorial model of interoceptive abilities, the measurement of interoceptive sensibility (representing ‘beliefs’) can be subdivided concerning interoceptive accuracy (e.g., confidence ratings) and interoceptive attention (e.g., self-reports such as the SBC), with the latter providing the most distinct findings in the current study. As the term ‘beliefs’ in a broader meaning has been adopted on the neural basis in the active inference framework, interoceptive dysfunctions might be characterized by overly strong expectations (or ‘beliefs’) shaping the perception of interoceptive signals [25]. When such expectations are not updated in case internal or external changes occur, resulting error signals prevail, further hindering adaptive homeostatic processes. One intriguing, yet speculative assumption is that patients with TCEs form interoceptive beliefs which (sub)-consciously disregard internal bodily experiences due to persistent internal error signals, which in turn leads to difficulties in emotion regulation. However, since interoceptive processes represent a complex cascade and further stress-mediating systems such as the immune system need to be investigated [83], the involved mechanisms mediating the impact of TCEs on interoception remain largely unaddressed, with the current study indicating altered interoceptive beliefs as a possible final result.

### Limitations

Several limitations should be acknowlegded: First, TCEs were assessed via self-report questionnaire. Although the CTQ self-report questionnaire has been shown excellent convergent validity with an clinical interview measure recently [104], subjective experiences of TCEs rather than actual exposure have been investigated as the low agreement between retrospective and prospective measures of TCEs indicates [105, 106]. Since the cross-sectional design does not allow for causal inferences, longitudinal studies are needed which investigate the association between interoception and prospective measures of TCEs.

Second, TCEs, body dissociation, and emotion dysregulation were all measured via self-report. Therefore, the observed relationship between solely self-report measures might be affected by monomethod bias. Moreover, body dissociation as measured by the SBC includes emotional disconnection [71], which might share at least some overlap with intolerance of distress as measured via the DERS [70]. Whether the observed relationships extend to other measures of body dissociation and emotion dysregulations needs to be adressed in future studies.

Third, due to the dimensional approach and matching rationale, HC and patients without the (self-reported) presence of TCEs were not investigated. By combining the groups, we were able to investigate and replicate a parallel mediation model of different interoceptive processes for the first time across individuals with TCEs in a large sample, thereby overcoming shortcomings of previous studies. Of note, patients with PTSD showed higher scores on the CTQ. Besides possible interaction effects of TCEs and clinical diagnosis, the HC group in the current study could be categorised as ‘resilient’ to a certain degree, in a sense that they adapted in the face of TCEs without developing a trauma-related disorder. Future studies are needed to investigate generalizability of the current findings.

Fourth, to the current state of knowledge, the validity of interoceptive accuracy based on the heartbeat counting task [72] is currently debated in the literature. Although reliability and convergent and discriminant validity have been recently investigated [107–109], comparisons between studies are difficult. Importantly, the results obtained in the current study were comparable to previous studies. However, future studies should adapt and compare different interoceptive tasks which incorporate different interoceptive organ systems and physical arousal states [87, 110–112], instead of the original heartbeat counting task.

Fifth, we did not control for sex, comorbidities, and medication which might have affected the results [92, 94, 98, 113–115]. In addition, future studies are needed which also examine the impact of TCEs on interoceptive processes in developmental disorders, since such disorders develop during sensitive time-periods during development wherein TCEs occur, which might limit generalizability of the current findings.

## Conclusion

TCEs represent an important risk factor for psychopathology such as emotion dysregulation, and might also impact certain interoceptive processes. The present findings confirmed self-reported body dissociation as a possible mediator between TCEs and emotion dysregulation. Developing psychotherapeutic interventions targeting interoceptive beliefs might prove to be a promising complement to existing interventions for patients affected by TCEs.

## Supplementary Information

**Additional file 1: Supplementary Table S1. **Current Comorbid and Lifetime Diagnoses of Mental Disorders and Psychotropic Load. **Supplementary Figure S1. **Parallel Mediation Model including Trait Dissociation in a Sample of Patients with Major Depression, Somatic Symptom Disorder, Posttraumatic Stress Disorder, and Healthy Controls (*N*=99).

## Data Availability

The data that support the findings of this study are available on reasonable request from the corresponding author.

## Abbreviations

ANS: Autonomic nervous system
BDI-II: Beck Depression Inventory revised
BPD: Borderline personality disorder
BSI: Brief Symptom Inventory
CTQ: Childhood Trauma Questionnaire
DERS: Difficulties in Emotion Regulation Scale
ECG: Electrocardiogram
FDS: German adaptation of the Dissociative Experience Scale (DES)
HB: Heartbeats
HC: Healthy controls
HRV: Heart rate variability
MD: Major depressive disorder
PCL-5: PTSD Checklist for DSM-5
PHQ-15: Patient Health-Questionnaire-15
PTSD: Posttraumatic stress disorder
RMSSD: Root-Mean-Square of Successive R–R-interval Differences
SAM: Sympatho-adreno-medullary
SBC: Scale of Body Connection
SCID-5: Structured Clinical Interview for DSM-5
SSD: Somatic symptom disorder
SSD-12: Somatic Symptom Disorder B-Criteria Scale
TCE: Traumatic childhood experience
