# Supplementary material for: The impact of traumatic childhood experiences on interoception: disregarding one’s own body
Source: Borderline Personal Disord Emot Dysregul. 2023 Feb 15;10:5. doi: 10.1186/s40479-023-00212-5 (PMC9930318; doi:10.1186/s40479-023-00212-5)
Supplement: Supplementary file 1 — Additional file 1: Supplementary Table S1. Current Comorbid and Lifetime Diagnoses of Mental Disorders and Psychotropic Load. Supplementary Figure S1. Parallel Mediation Model including Trait Dissociation in a Sample of Patients with Major Depression, Somatic Symptom Disorder, Posttraumatic Stress Disorder, and Healthy Controls (N=99). [file 40479_2023_212_MOESM1_ESM.docx]

**Supplementary Information**

**Supplementary sample characteristics**

Current comorbid and lifetime mental disorders, and psychotropic medication load are depicted in Table S1.

Current psychotropic medication was restricted to regular medication with antidepressants, antipsychotics (sleep-inducing effect only) and anticonvulsants (i.e., pregabalin, pain-relieving effect only) for patients with mental disorders. Psychotropic medication load was calculated as a composite measure following procedures outlined in previous studies [1-3]. Daily dosages for each medication were coded as absent=0, low-dose=1, or high-dose=2. The composite measure was calculated as the sum score of number and dosages of all medications taken by an individual participant. Antidepressants were categorized as low- (levels 1 and 2) or high-dose (levels 3 and 4) [4]. Chlorpromazine dose equivalents were used for scoring of antipsychotic medication. Chlorpromazine dose equivalents equal or below, or above the mean effective daily dose of chlorpromazine represent low- or high-dosings, respectively [5, 6]. Pregabalin dosing was coded as low (≤300 mg) or high (>300 mg) [7, 8].

**Supplementary Table S1**

*Current Comorbid and Lifetime Diagnoses of Mental Disorders and Psychotropic Load*

|  | MD  (*n*=35)  (24 women) | SSD  (*n*=34)  (26 female) | PTSD  (*n*=33)  (28 female) | HC  (*n*=34)  (27 female) |
| --- | --- | --- | --- | --- |
| Current comorbid Diagnoses^a^ |  |  |  |  |
| MD | 35 (35) | 7 (21) | 8 (24) | 0 (0) |
| SSD | 1 (1) | 34 (34) | 2 (4) | 0 (0) |
| PTSD | 2 (3) | 1 (1) | 33 (33) | 0 (0) |
| Other affective disorders | 3 (2) | 2 (3) | 3 (3) | 0 (0) |
| Other somatization disorders | 0 (0) | 0 (0) | 0 (0) | 0 (0) |
| Other anxiety disorders | 8 (10) | 4 (7) | 5 (9) | 0 (0) |
| Eating disorders | 2 (4) | 1 (7) | 3 (9) | 0 (0) |
| Substance use disorders | 0 (5) | 0 (5) | 0 (12) | 0 (0) |
| Psychotropic medication load^b^ | 1.31 (1.37) | 0.50 (1.02) | 0.76 (1.00) | 0.00 (0.00) |

*Note.* HC, healthy controls; MD, major depressive disorder; PTSD, posttraumatic stress disorder; SD, standard deviation; SSD, somatic symptom disorder.

^a^ Data are presented as current (lifetime) diagnosis

^b^ Data are presented as mean (SD)

**Supplementary information on Materials and Methods**

*Traumatic childhood experiences*

The *Childhood Trauma Questionnaire* (CTQ) [9] assesses *traumatic childhood experiences* (TCEs). Participants rate frequency of TCEs on a 5-point scale (ranging from ‘never true’ to ‘very often true’) for the five scales *physical* (e.g., ‘People in my family hit me so hard that it left me with bruises or marks’), *sexual* (e.g., ‘Someone molested me’), and *emotional abuse* (e.g., ‘I thought that my parents wished I had never been born’), and *physical* (e.g., ‘I had to wear dirty clothes’) and *emotional neglect* (e.g., ‘I felt loved’ [reverse coded]) with five items each (resulting in corresponding scores from 5 to 25). A total sum score can be calculated from the scales, ranging from 25 to 125 (overall internal consistency *α*=.90).

*Psychopathology*

Specifically trained diagnosticians assessed mental health disorders using the German version of the *Structured Clinical Interview for DSM-5* (SCID-5) [10]. Interrater reliability was based on 12 randomly selected video-taped diagnostic interviews rated by five independent raters and the head of the diagnostic unit, and yielded an excellent [11] score of κ=1.00.

The *Patient Health-Questionnaire-15* (PHQ-15) [12] was used to assess the presence and severity of common somatic symptoms within the last 4 weeks using 15 items. Each symptom can be scored from 0 (‘not bothered at all’) to 2 (‘bothered a lot’) with sum scores ranging from 0 to 30 (overall internal consistency α=.82).

The *Somatic Symptom Disorder - B Criteria Scale* (SSD-12) [13] consists of 12 items, with 4 items for each of the three psychological sub-criteria comprising cognitive, affective and behavorial aspects. Item scores range between 0 (‘never’) and 4 (‘very often’) and the total sum score ranges between 0 and 48 (overall internal consistency α=.94).

The *Posttraumatic Stress Disorder Checklist for DSM-5* (PCL-5) [14] was used to assess the presence and severity of PTSD symptoms over the past month. Item scores range between 0 (‘not at all’) and 4 (‘extremely’) and sum scores the total sum score ranges between 0 and 80 (overall internal consistency α=.95).

Severity of depressive symptoms was assessed with the *Beck-Depression-Inventory II* (BDI-II) [15]. Each of the 21 items represents an affective or somatic symptom related to depression, with statement scales ranging from 0 (‘no disturbance’) to 3 (‘maximal disturbance’), and corresponding sum scores ranging from 0 to 63 (overall internal consistency α=.95).

General symptom severity was assessed with the *Brief Symptom Inventory* (BSI) [16]. Nine primary symptom dimensions are measured with 53 items ranging from 0 ‘not at all’ to 4 ‘extremely’. In the current study, the *BSI Global Severity Index* (BSI GSI), which is the mean score of all items, was used (overall internal α=.96).

The German adaptation of the Dissociative Experience Scale, that is, the Fragebogen zur Erfassung Dissoziativer Symptome (FDS) [17, 18] was used to assess trait dissociation. The FDS consists of 44 items measuring the frequency of dissociative experiences (in 10% increments, ranging from 0 to 100) on the dimensions amnesia (e.g., ‘Some people find evidence that they have done things that they do not remember doing’), absorption/imaginative involvement (e.g., ‘Some people have the experience of not being sure whether things that they remember happening really did happen or whether they just dreamed them’), derealisation/depersonalization (e.g., ‘Some people sometimes have the experience of feeling that other people, objects, and the world around them are not real’) and conversion (e.g., ‘Some people sometimes have difficulties with their eyes, e.g., double or blurred vision, blind in one or both eyes, without a doctor being able to find a physical cause’). A mean score can be calculated, ranging from 0 to 100 (internal consistency in the present study *α*=.94).

*Emotion dysregulation*

The *Difficulties in Emotion Regulation Scale* was used to assess emotion regulation deficits (DERS) [19]. The DERS comprises six subscales: *nonacceptance of negative emotions* (6 items; e.g., ‘When I’m upset, I become angry with myself for feeling that way’), *difficulties engaging in goal-directed behaviors when distressed* (5 items; e.g., ‘When I’m upset, I have difficulty focusing on other things’), *difficulties controlling impulsive behaviors when distressed* (6 items; e.g., ‘When I’m upset, I become out of control‘), *limited access to effective emotion regulation strategies* (8 items; e.g., ‘When I’m upset, I believe there is nothing I can do to make myself feel better’), *lack of emotional awareness* (6 items; e.g., ‘When I’m upset, I acknowledge my emotions’ [reverse coding]) and *lack of emotional clarity* (5 items; e.g., ‘I have difficulty making sense out of my feelings’) [20]. Participants rate each item on a 5-point scale ranging from 1 (‘almost never’) to 5 (‘almost always’). A total sum score can be calculated, ranging from 36 to 180 (overall internal consistency *α*=.94).

*Interoceptive sensibility*

The *Scale of Body Connection* (SBC) [21] was used to assess self-reported *body awareness* and *body dissociation* during the last two months. The subscale *body awareness* (12 items; overall internal consistency *α*=.79) measures attention to bodily signals in everyday situations and the perception of bodily responses to emotions (e.g., ‘I take cues from my body to help me understand how I feel’). The subscale *body dissociation* (8 items; (overall internal consistency *α*=.80) refers to the avoidance or disregard of internal bodily experiences and the feeling of seperatedness from one’s own body (e.g., ‘I distract myself from feelings of physical discomfort’). The SBC has been proposed to reflect confidence in interoceptive ability and preferential or habitual attendance to interoceptive signals [22], which has later been incorporated in the *Multidimensional Assessment of Interoceptive Awareness* (MAIA) [23] a further well-established measure of interoceptive sensibility. Each item of the SBC is scored on a 5-point scale, ranging from 0 ‘not at all’ to 4 ‘all of the time’, with higher values indicating higher body awareness and body dissociation, respectively.

*Additional measures*

Demographic details, height, and weight were assessed with a standardized questionnaire. Education was assessed based on years completed in the stardard German school system (9 years=certificate of secondary education, 10 years=general certificate of secondary education, 13 years=university entrance diploma).

**Supplementary information on Procedure**

*Heartbeat counting task*

Participants were instructed to sit still and relaxed in a sound-attenuated, dimly lit room for a 5-minute resting-state electrocardiogram (ECG) measurement. After the resting state measurement, participants were asked to silently count their own heartbeats without manually checking. Start and stop times were defined by an acoustic signal. After two practice trials of 30 seconds, the task was repeated in seven consecutive time intervals (20, 25, 35, 45, 55, 65, 75 seconds each), whereby the duration of the time intervals was unknown to the participants. After each time interval, participants were asked to indicate the number of counted heartbeats via keyboard. In addition, participants were asked to judge how confident they were with regard to their own counting abilities (‘How well do you think you perceived your heartbeat?’ with scores ranging between 1 (‘not at all’) and 9 (‘very well’)).

*Additional information on ECG Recording and Processing*

For the electrocardiogram, three skin electrodes (Red DotTM Ag/AgCl, Micropore tape and solid gel, 3 M Health Care), were attached to the front of the subject's body (right anterior: below the right collarbone, left low: left bottom centered on the 7th rib, right low: right lower abdomen) and a reference electrode (standard ECG electrode, Ag/AgCl) under the right shoulder opposite the right anterior electrode. The electrodes had a diameter of 45mm and contained an inner gel layer with a diameter of 2mm. The ECG was recorded using an AccuSync® 71 trigger monitor in combination with a QuickAmp Amplifier (Brain Products GmbH; pass-band filter: 0.01-200 Hz; sampling rate: 1000 Hz) for the reference electrode. The signal was recorded with a differential amplifier and a high-pass filter at 0.5Hz for offline analysis. The R-spikes for pulse detection were identified online via the AccuSync® 71 monitor and BrainVision Recorder 1.20 and offline using an ECG marker macro and manual inspection in the BrainVision Analyzer 2.1.

For HRV-Analysis, heart rate (HR) and R-peaks were detected offline with EDF-Browser Software employing combined adaptive thresholding [24], followed by a visual inspection for artifacts and correct R-trigger location. Inter-beat-intervals (IBI) were then computed and further processed using the RHRV package, a widely used HRV analysis in R [25]. Cleaned and processed IBIs were additionally visually reviewed by the researcher for artifacts and, in case of presence, manually removed. In a final step, RMSSD indices were derived automatically and transferred to SPSS (IBM SPSS 26). RHRV-extracted HRV-parameters show high reliability and validity compared to respective parameters as retrieved by Kubios (*r*s > 0.8), a “gold standard software” for HRV analysis [26].

**Mediation analysis**

In order to further validate the mediating role of body dissociation, trait dissociation (FDS total score) was included in the mediation model as presented in the manuscript. Including trait dissociation as parallel mediator did not change the pattern of results (*b*=0.260, 95% CI [0.098, 0.518] for body dissociation and b=0.045, 95% CI [-0.023, 0.252] for trait dissociation). Again, the total effect was significant (*b*=0.341, *p*=.017), whereas the direct effect was statistically not significant (*b*=0.030, *p*=.808, adjusted robust *R*^2^=.343), suggesting that body dissociation fully mediated the association between traumatic childhood experiences and emotion dysregulation even after controlling for trait dissociation (see Fig. S1).

**Supplementary Figure S1**

*Parallel Mediation Model including Trait Dissociation in a Sample of Patients with Major Depression, Somatic Symptom Disorder, Posttraumatic Stress Disorder, and Healthy Controls (N=99)*


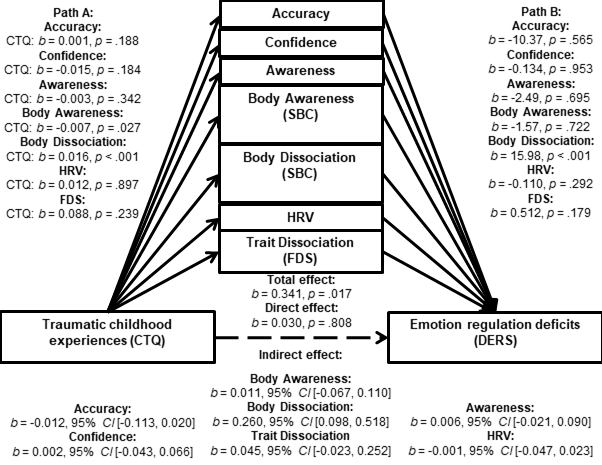


*Note.* Path A represents the effect of the predictor on each mediator, path B represents the combined relationship of each mediator with the outcome, with the direct effect representing the effect of the predictor on the outcome after inclusion of all mediators and the total effect representing the basic relationship between the predictor and the outcome. The indirect effect represents the combined effect of path A and path B and therefore the mediation. Significance inferences at the 0.05 α level are based upon the notion whether confidence intervals include zero.

*Abbreviations.* CTQ, Childhood Trauma Questionnaire; DERS, Difficulties in Emotion Regulation Scale; FDS, German adaptation of the Dissociative Experience Scale; HRV, heart rate variability; SBC; Scale of Body Connection

**eReferences**

1. Hassel S, Almeida JR, Kerr N, Nau S, Ladouceur CD, Fissell K, et al. Elevated striatal and decreased dorsolateral prefrontal cortical activity in response to emotional stimuli in euthymic bipolar disorder: no associations with psychotropic medication load. Bipolar Disord. 2008;10(8):916-27. <https://doi.org/10.1111/j.1399-5618.2008.00641.x>

2. Schwarz K, Moessnang C, Schweiger JI, Baumeister S, Plichta MM, Brandeis D, et al. Transdiagnostic Prediction of Affective, Cognitive, and Social Function Through Brain Reward Anticipation in Schizophrenia, Bipolar Disorder, Major Depression, and Autism Spectrum Diagnoses. Schizophr Bull. 2020;46(3):592-602. <https://doi.org/10.1093/schbul/sbz075>

3. Eckstrand KL, Forbes EE, Bertocci MA, Chase HW, Greenberg T, Lockovich J, et al. Anhedonia Reduction and the Association Between Left Ventral Striatal Reward Response and 6-Month Improvement in Life Satisfaction Among Young Adults. JAMA Psychiatry. 2019;76(9):958-65. <https://doi.org/10.1001/jamapsychiatry.2019.0864>

4. Sackeim HA. The definition and meaning of treatment-resistant depression. Journal of Clinical Psychiatry. 2001;62:10-7.

5. Gardner DM, Murphy AL, O'Donnell H, Centorrino F, Baldessarini RJ. International consensus study of antipsychotic dosing. Am J Psychiatry. 2010;167(6):686-93. <https://doi.org/10.1176/appi.ajp.2009.09060802>

6. Leucht S, Samara M, Heres S, Davis JM. Dose Equivalents for Antipsychotic Drugs: The DDD Method. Schizophr Bull. 2016;42 Suppl 1:S90-4. <https://doi.org/10.1093/schbul/sbv167>

7. Kasper S, Iglesias-Garcia C, Schweizer E, Wilson J, DuBrava S, Prieto R, et al. Pregabalin long-term treatment and assessment of discontinuation in patients with generalized anxiety disorder. Int J Neuropsychopharmacol. 2014;17(5):685-95. <https://doi.org/10.1017/S1461145713001557>

8. Serpell M, Latymer M, Almas M, Ortiz M, Parsons B, Prieto R. Neuropathic pain responds better to increased doses of pregabalin: an in-depth analysis of flexible-dose clinical trials. J Pain Res. 2017;10:1769-76. <https://doi.org/10.2147/JPR.S129832>

9. Bernstein DP, Stein JA, Newcomb MD, Walker E, Pogge D, Ahluvalia T, et al. Development and validation of a brief screening version of the Childhood Trauma Questionnaire. Child Abuse Negl. 2003;27(2):169-90. <https://doi.org/10.1016/s0145-2134(02)00541-0>

10. Beesdo-Baum K, Zaudig M, Wittchen HU. *SCID-5-CV. Strukturiertes Klinisches Interview für DSM-5®-Störungen – Klinische Version. Deutsche Bearbeitung des Structured Clinical Interview for DSM-5® Disorders – Clinician Version von Michael B. First, Janet B. W. Williams, Rhonda S. Karg, Robert L. Spitzer.[SCID-5-CV. Structured Clinical Interview for DSM-5® disorders – Clinical Version. German adaptation of the Structured Clinical Interview for DSM-5 disorders – Clinician Version by Michael B. First, Janet B. W. Williams, Rhonda S. Karg, Robert L. Spitzer]*. Hogrefe.; 2019.

11. Cicchetti D. Guidelines, criteria, and rules of thumb for evaluating normed and standardized assessment instruments in psychology. Psychol Assess. 1994;6(4):284-90. <https://doi.org/doi:10.1037/1040-3590.6.4.284>

12. Kroenke K, Spitzer RL, Williams JB. The PHQ-15: validity of a new measure for evaluating the severity of somatic symptoms. Psychosom Med. 2002;64(2):258-66. <https://doi.org/10.1097/00006842-200203000-00008>

13. Toussaint A, Lowe B, Brahler E, Jordan P. The Somatic Symptom Disorder - B Criteria Scale (SSD-12): Factorial structure, validity and population-based norms. J Psychosom Res. 2017;97:9-17. <https://doi.org/10.1016/j.jpsychores.2017.03.017>

14. Kruger-Gottschalk A, Knaevelsrud C, Rau H, Dyer A, Schafer I, Schellong J, et al. The German version of the Posttraumatic Stress Disorder Checklist for DSM-5 (PCL-5): psychometric properties and diagnostic utility. BMC Psychiatry. 2017;17(1):379. <https://doi.org/10.1186/s12888-017-1541-6>

15. Hautzinger M, Bailer M, Worall H, Keller F. Beck-Depressionsinventar (BDI): Bearbeitung der deutschen Ausgabe. Testhandbuch. Bern: Hans-Huber; 1994.

16. Derogatis LR, Melisaratos N. The Brief Symptom Inventory: an introductory report. Psychol Med. 1983;13(3):595-605.

17. Spitzer C, Freyberger HJ, Stieglitz RD, Carlson EB, Kuhn G, Magdeburg N, et al. Adaptation and psychometric properties of the German version of the Dissociative Experience Scale. J Trauma Stress. 1998;11(4):799-809. <https://doi.org/10.1023/A:1024457819547>

18. Freyberger HJ, Spitzer C, Stieglitz RD. Fragebogen zu dissoziativen Symptomen FDS. Bern: Huber; 1999.

19. Gratz KL, Roemer L. Multidimensional assessment of emotion regulation and dysregulation: Development, factor structure, and initial validation of the difficulties in emotion regulation scale. J Psychopathol Behav Assess. 2004;26(1):41-54.

20. Fossati A, Gratz KL, Somma A, Maffei C, Borroni S. The Mediating Role of Emotion Dysregulation in the Relations Between Childhood Trauma History and Adult Attachment and Borderline Personality Disorder Features: A Study of Italian Nonclinical Participants. J Pers Disord. 2016;30(5):653-76. <https://doi.org/10.1521/pedi_2015_29_222>

21. Price CJ, Thompson EA. Measuring dimensions of body connection: body awareness and bodily dissociation. J Altern Complement Med. 2007;13(9):945-53. <https://doi.org/10.1089/acm.2007.0537>

22. Khoury NM, Lutz J, Schuman-Olivier Z. Interoception in Psychiatric Disorders: A Review of Randomized, Controlled Trials with Interoception-Based Interventions. Harv Rev Psychiatry. 2018;26(5):250-63. <https://doi.org/10.1097/HRP.0000000000000170>

23. Mehling WE, Price C, Daubenmier JJ, Acree M, Bartmess E, Stewart A. The Multidimensional Assessment of Interoceptive Awareness (MAIA). PLoS One. 2012;7(11):e48230. <https://doi.org/10.1371/journal.pone.0048230>

24. Christov, II. Real time electrocardiogram QRS detection using combined adaptive threshold. Biomed Eng Online. 2004;3(1):28. <https://doi.org/10.1186/1475-925X-3-28>

25. Martínez CAG, Quintana AO, Vila XA, Touriño MJL, Rodríguez-Liñares L, Presedo JMR, et al. Heart rate variability analysis with the R package RHRV. Cham, Switzerland: Springer International Publishing; 2017.

26. Zhang N, Hoch J, Gewirtz AH. The Physiological Regulation of Emotion During Social Interactions: Vagal Flexibility Moderates the Effects of a Military Parenting Intervention on Father Involvement in a Randomized Trial. Prev Sci. 2020;21(5):691-701. <https://doi.org/10.1007/s11121-020-01122-6>
